# O15 - Component resolved diagnosis: performance of specific IgE to alternaria compared to Alt a 1

**DOI:** 10.1186/2045-7022-4-S1-O15

**Published:** 2014-03-14

**Authors:** Isabel Lafuente, Raquel Pina, Sonia Uixera, Rafael Calderon, Isidoro Cortell, Juan Lopez, Antonio Nieto, Angel Mazon

**Affiliations:** 1 Unit of Pediatric Allergy and Pneumology Hospital La Fe, Valencia, Spain

## Objective

As the panel of molecules available for component resolved diagnosis is being expanded our objective was to compare the performance of specific IgE to Alt a 1 and to Alternaria.

## Material and methods

Specific IgE (ImmunoCAP^®^, Thermo Fisher Scientific) to Alternaria (cost ≈ 8 -9 €) and to Alt a 1 (cost ≈ 11-12 €) were measured simultaneously in children with symptoms of asthma and/or rhinoconjunctivitis who had positive skin tests to Alternaria. Values <0.35 kUA/L were considered negative.

## Results

We studied 143 children (92 M/51 F) aged 2.4 - 15.6 years. Negative results were found in 20 (14%) with Alternaria, 24 (16.8%) with Alt 1, and 19 with both (13.3%). There was a good correlation between the two determinations (r = 0.935, p <0.001). Overall the levels were higher for Alt a 1 (18.9 ± 24.4) than for Alternaria (15.4 ± 20.1). There were 97 cases (68%) with levels of Alt a 1 > Alternaria, 42 (29%) with Alt a 1 < Alternaria, and four with the same values. There was a single patient with positive Alt a 1 (0.39) and negative Alternaria (0.3), and five patients with negative Alt a 1 and positive Alternaria, with values of 0.43, 0.56, 2.3 , 7.4 and 11.3, two of which are not considered very meaningful.

## Conclusion

The performance of specific IgE against Alternaria and Alt a 1 in detection of positive cases is similar. Only 2% of the cases appear to be sensitized to other molecules different from Alt a 1, and could go unnoticed if only the latter determination is requested. On the other hand, current vaccines standardized in biological units are based on whole Alternaria, and those in weight/volume are based on Alt a 1.

